# Effects of Annealing Temperature and Time on Properties of Thermoplastic Polyurethane Based on Different Soft Segments/Multi-Walled Carbon Nanotube Nanocomposites

**DOI:** 10.3390/polym15020364

**Published:** 2023-01-10

**Authors:** Kittimon Jirakittidul, Darawan Limthin, Sarita Mahithithummathorn, Seenam Phaewchimphlee

**Affiliations:** Department of Chemistry, School of Science, King Mongkut’s Institute of Technology Ladkrabang, Bangkok 10520, Thailand

**Keywords:** annealing, multiwall carbon nanotube, thermoplastic polyurethane, electrical conductivity

## Abstract

Typically, polymer chains can move under the annealing process, resulting in an ordered structure arrangement. This causes an improvement in nanocomposite properties and in the dispersion of filler. In this research, annealed thermoplastic polyurethane (PU)/multi-walled carbon nanotube (MWCNT) nanocomposites were studied to investigate the effect of annealing on the selective dispersion of MWCNTs. PU matrices were composed of two different soft segments, i.e., polyether (PU-Ether) and polyester (PU-Ester). Nanocomposites were prepared by the melt mixing process and annealed at 80 to 120 °C for 6 to 24 h. The increases in annealing time and temperature resulted in microphase separation in segmented PU and the orientation of crystalline structures in the segregated hard domain. Nanocomposites showed higher electrical conductivity after annealing. This implies that the movement of PU chains during heat treatment encouraged the development of the MWCNT network. However, the increase in ordered structures could obstruct the MWCNT network, resulting in lower electrical conductivity levels. Considering the selective dispersion of MWCNT in PU matrices, it was found that MWCNTs dispersed in soft segments of PU-Ether, leading to a significant decrease in elongation at the break after annealing. On the other hand, a decrease in elasticity of PU-Ester nanocomposites was not observed as a result of MWCNT dispersal in hard segments.

## 1. Introduction

One of the most attractive one-dimensional nanomaterials is the carbon nanotube. This material exhibits excellent combination properties, specifically electrical conductivity, mechanical properties and thermal stability. There are two main types of carbon nanotube: single-walled carbon nanotubes (SWCNT) and muti-walled carbon nanotubes (MW CNT). MWCNTs are arrays of graphene cylinders with an outer diameter of between 2.5 and 30 nm [1]. As a result of their high aspect ratio and high surface energy, they can entangle and form bundle agglomerates with Van der Waals forces [1,2,3,4,5]. In order to achieve superior nanocomposite properties, MWCNTs are necessary to achieve good dispersion in the polymer matrix.

Typically, thermoplastic PU is a block copolymer which consists of hard and soft segments with urethane linkage (-NHCO-O-) within their molecular structures. Hard segments (containing urethane linkage) are rigid chains, but soft segments are flexible chains. The range of possible chemical structures for the soft segments in segmented PUs provides a broad range of properties due to variations in chain flexibility and the degree of interaction between the hard and soft segments. Differences in separation and domain size lead to variations in thermal behaviors and mechanical properties [6]. The most frequently used soft segments for PU are polyether or polyester polyols. Typically, the hydrogen bonding between ester carbonyl groups and N-H groups in the hard segments is stronger than the interaction of ether oxygen groups, with N-H groups leading to more microphase separation in PU based on polyether [7]. Consequently, PU based on polyesters provides superior physical properties. However, PU based on polyether shows higher hydrolysis resistance and greater low-temperature properties [7,8,9]. Moreover, the optical and adhesive properties of segmented polyurethanes based on different soft segments (i.e., polyether and polycarbonate) were investigated by Puszka and Kultys [10]. Polyurethane, based on polycarbonate, showed superior transparency and adhesive properties. The compatibility of highly polar hard segments and less polar soft segments is limited, leading to microphase separation [11,12,13]. The properties of PU are evaluated in terms of the segregated PU phase results. The soft-segment domain shows elastomeric properties. On the other hand, the hard-segment domain forms hydrogen bonding with the urethane functional group and acts as a physical crosslink within the structure.

The properties of nanocomposites are not only affected by the nature of the chemical structure of the segmented PU but also by thermal history. Annealing is a heat treatment process which is interesting for improving the polymer nanocomposite properties of various polymer types [14,15,16,17,18,19,20,21,22,23,24,25]. The annealing process uses a traditional machine; it is, therefore, familiar to industrial manufacturing and useful for developing nanocomposite properties with economic benefits. Polymer chains are able to move and rearrange themselves into an ordered structure when heated to temperatures between glass transition and melting temperature. However, the crystallization of a polymer matrix introduced by heat treatment is able to either encourage or obstruct the formation of a conductive network of carbon nanotubes [16]. Block copolymer, particularly thermoplastic PU, is able to introduce microphase separation after undergoing the annealing process [12,13,26,27,28,29]. When PU/carbon nanotube nanocomposites were annealed, their conductivity and mechanical properties were improved [17,18,19]. In this research, annealing treatment was applied to improve the microphase separation of segregated PU and to develop selective MWCNT dispersion in segregated PU domains. The effects of annealing temperature and time were also studied.

## 2. Materials and Methods

### 2.1. Materials

MWCNTs were purchased from Sigma Aldrich. The inner and outer diameters were 4.5 and 10 nm, respectively. The length of nanotubes was 4 μm, with an aspect ratio (L/D) of 350–550 [30]. PU-Ether (Utechllan^®^ UE-95AU) and PU-Ester (Desmopan^®^ 3695AU DPS101), based on methylene diphenyl diisocyanates (MDI) hard segments, were purchased from Bayer, Taiwan.

### 2.2. Fabrication of Nanocomposite

MWCNT and PU were dried in an oven to eliminate moisture. PU/MWCNT nanocomposites at 0.5%wt were prepared via melt mixing using a Brabender Plasti-Corder internal mixer, model PL2100, with a rotation speed of 60 rpm at a temperature of 200 °C for 10 min and 210 °C for 8 min for PU-Ester and PU-Ether, respectively. Following this, nanocomposites were shaped into flat sheets with a dimension of 10 × 10 cm^2^ and a thickness of 1 mm in between polytetrafluoroethylene sheets by compression molding at a temperature of 200 °C for 8 min.

### 2.3. Annealing Process

All compressed nanocomposite sheets were annealed at 80, 100 and 120 °C for 6, 12 and 24 h in an oven. They were named a-b-c; a was a soft-segment PU structure (Et for PU-Ether and Es for PU-Ester), b was annealing temperature and c was annealing time. For example, Et-120-24 was PU-Ether nanocomposite, annealed at 120 °C for 24 h.

### 2.4. Characterization

Thermal behaviors of nanocomposites were measured by differential scanning calorimetry (DSC). A Netzsch DSC 204 F1 Phoenix^®^, Germany was used. The range of temperatures studied was from −70 °C to 250 °C, with a heating rate of 10 °C·min^−1^ under nitrogen gas. A universal testing machine from Cometech, Taiwan was used to study mechanical properties. Tensile testing followed ASTM D638 at a crosshead speed of 500 mm·min^−1^. To investigate MWCNT dispersion in PU matrices, the fracture surfaces of nanocomposites undergoing tensile testing were investigated by a field emission scanning electron microscope (FESEM), using the model JSM-7610F obtained from Jeol, Japan. The samples were coated with Pt. The accelerating voltage of the electron beam was set at 5 kV. The resistance of PU/MWCNT nanocomposite at a dimension of 1 × 1 cm^2^ was determined using an Agilent E4980A precision LCR meter set at 20–1 × 10^6^ Hz. The volume conductivities of all nanocomposites were calculated to study electrical properties.

## 3. Results and Discussion

### 3.1. Thermal Behavior

**Figure 1:**
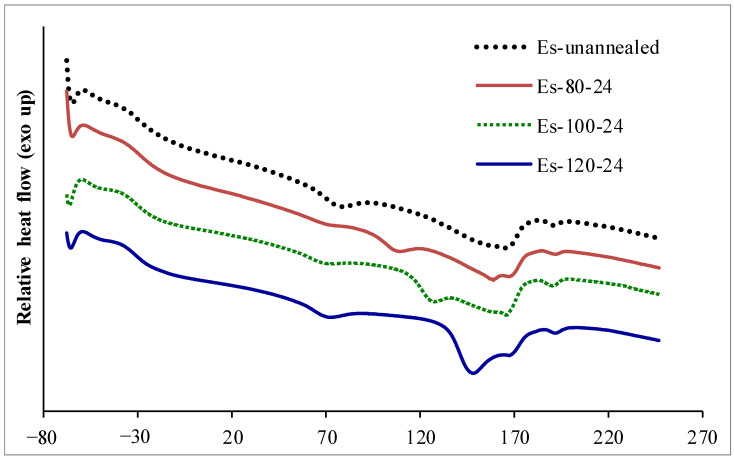
DSC thermographs of PU-Ester/MWCNT nanocomposites annealed under different conditions.

**Figure 2:**
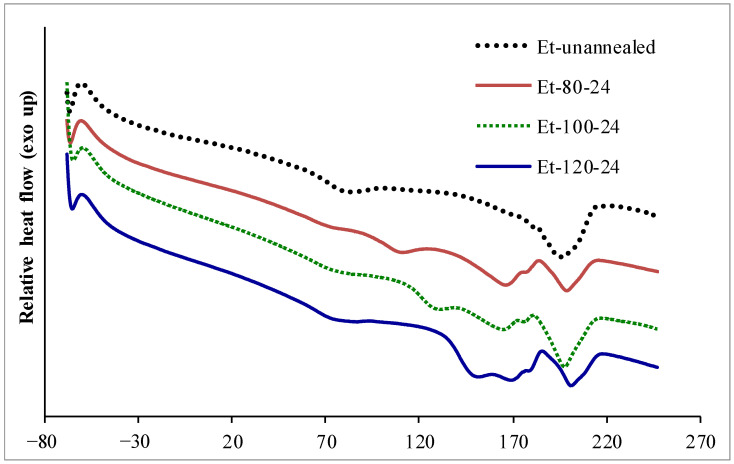
DSC thermographs of PU-Ether/MMCNTs nanocomposites.

DSC is a useful technique to characterize the thermal properties of a block copolymer hieratical structure, particularly thermoplastic PU [11,12,13,27,28,29]. Nanocomposites were characterized by DSC to study microphase separation of segmented PU and the orientation of crystalline material in the segregated hard phase. DSC thermographs of PU-Ester nanocomposites and PU-Ether nanocomposites are shown in Figure 1 and Figure 2. Because the block copolymer structure of thermoplastic PU consists of soft and hard segments, the difference in polarity of these two segments leads to limited compatibility and microphase separation. Thermographs showing glass transition temperature of segregated soft phase (T_g(SP)_), glass transition temperature of mixing phase between soft and hard segments (T_g(MP)_) and microphase mixing temperature (T_MM_) and enthalpy of microphase mixing peak (ΔH_MM_) were analyzed, as shown in Table 1. It was observed that the microphase mixing endothermic peak split into two peaks: at a lower temperature range (between 120 °C and 180 °C), termed T_MM(L)_, and at a higher temperature range (higher than 180 °C), termed T_MM(H)_. Moreover, multiple tops were found on microphase mixing endothermic peaks at the lower temperature range; they were, therefore, named following the increment of temperature found (i.e., A, B and C). The effects of the annealing process on multiple melting endotherms of segmented PU were also studied by several researchers [12,13,26,27]. Saini et al. [12,13] investigated the effects of the annealing temperature and time on thermoplastic PU by using DSC. Two melting endotherms were found after undergoing the annealing process; a lower-temperature melting endotherm, related to the melting of ordered structures in the segregated hard domain, and a higher-temperature melting endotherm. With increased annealing temperature, these melting temperatures and enthalpies increased. Furthermore, it was suggested that the effects of annealing firstly increased the microphase separation and subsequently increased the ordering of the segregated hard phase.

PU-Ester nanocomposites without annealing showed T_g_(_SP_) at −30 °C, T_g_(_MP_) at 69 °C and two microphase mixing peaks: T_MM_(_L_) at 165 °C and T_MM_(_H_) at 191 °C with ΔH_MM_ of 14.9 J·g^−1^. Thermal transitions of unfilled PU-Ester were characterized. T_g_(_SP_) at −25 °C, T_g_(_MP_) at 68 °C and T_MM_ at 162 °C and T_MM_(_H_) at 191 °C, with ΔH_MM_ of 14.6 J·g^−1^, were found. Annealed PU-Ester nanocomposites showed lower T_g_(_SP_) and T_g_(_MP_), indicating that the annealing process induced the microphase separation of soft and hard phases. T_g_(_HP_) was found at approximately 100 °C on an annealing temperature of 80 °C, indicating the amorphous state of the segregated hard segment phase. With increased annealing temperature, T_MM_(_L_)_A_ was observed in the range of 125–149 °C; at an annealing temperature of 120 °C, T_MM_(_L_)_A_ was approximately 20 °C higher than at an annealing temperature of 100 °C. Higher enthalpy of microphase mixing was also observed after annealing. These contributed to the higher annealing temperature accommodating the movement of the segregated hard-segment domain to the orientation of the ordered structure, resulting in higher melting enthalpy for the dissociation of hydrogen bonding. Therefore, the annealing associated the segregated hard domain to align the amorphous form and subsequently crystallize with an annealing temperature increase.

For the unannealed PU-Ether nanocomposites in Table 1, T_g_(_MP_) at 74 °C, T_MM_(_H_) at 196 °C and ΔH_MM_ at 14.9 J·g^−1^ were found. Unfilled PU-Ether showed T_g_(_MP_) at 70 °C and T_MM_ at 162 °C and two microphase mixing peaks: T_MM_(_L_) at 174 °C and T_MM_(_H_) at 183 °C with ΔH_MM_ of 15.1 J·g^−1^. The annealing also introduced microphase separation with regard to T_g_(_MP_), which decreased by 6 to 12 °C. For PU-Ether nanocomposites, annealed at 80 and 100 °C, T_g_(_HP_) were found to have increased by 20 °C with increasing annealing temperature. At an annealing temperature of 120 °C, T_g_(_HP_) was not found, but T_MM_(_L_)_A_ was observed and increased with annealing time. These results revealed that hard segments in PU-Ether were able to move and align in amorphous form at annealing of 80 to 100 °C. The orientation of the ordered structure in crystalline form was introduced at 120 °C. Comparing the PU-Ester and PU-Ether nanocomposites, it was found that the crystallization of segregated PU-Ether hard segments occurred at higher annealing temperatures. It is possible that PU-Ether nanocomposites had a higher microphase mixing temperature and higher melt viscosity.

### 3.2. Mechanical Property

**Figure 3:**
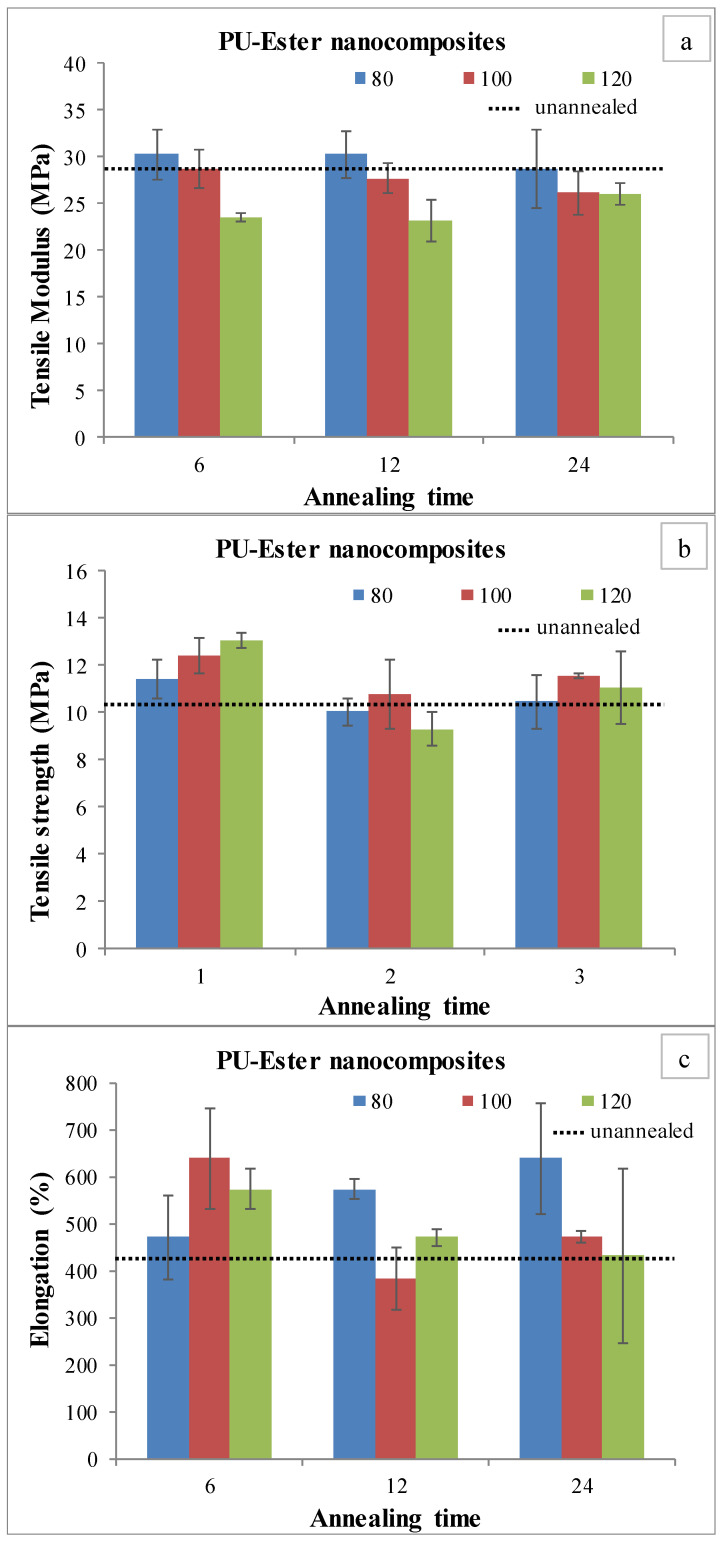
Tensile testing of annealed PU-Ester/MWCNT nanocomposites; tensile modulus (**a**), tensile strength (**b**) and elongation at break (**c**).

**Figure 4:**
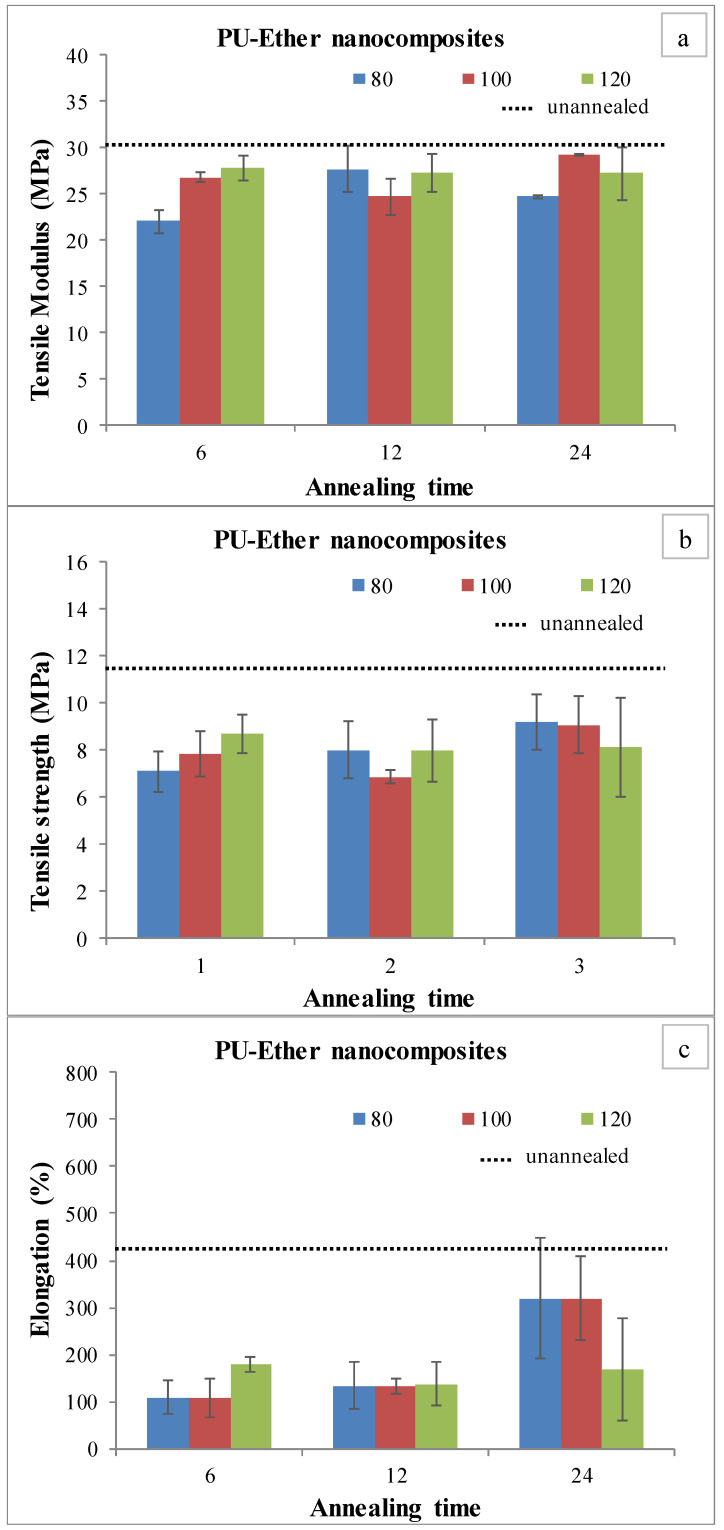
Tensile testing of annealed PU-Ether/MWCNT nanocomposites; tensile modulus (**a**), tensile strength (**b**) and elongation at break (**c**).

Nanocomposites were characterized by a tensile testing machine to investigate tensile strength, modulus and elongation at break. The mechanical properties of PU-Ester and PU-Ether nanocomposites are shown in Figure 3 and Figure 4, respectively. Unannealed PU-Ester nanocomposite had a tensile strength of 10.4 ± 1.0 MPa, a tensile modulus of 28.8 ± 1.2 MPa and an elongation of 429 ± 99%. For unannealed PU-Ether nanocomposites, tensile strength was found to be 11.5 ± 1.3 MPa, tensile modulus was 30.5 ± 1.9 MPa and elongation was 425 ± 175%.

Considering annealed PU-Ester nanocomposites, the tensile modulus tended to decrease as annealing temperature increased. On the other hand, tensile strength and elongation developed. It is possible that MWCNTs preferably dispersed in the segregated hard domain. The soft segments were able to be flexible, meaning they were not influenced by the decrease in elasticity of the PU-Ester nanocomposites. However, decreases in mechanical properties were found after the PU-Ether/MWCNT nanocomposites had been treated by heat. It was found that tensile strengths and elongation at break decreased significantly, as shown in Figure 4b,c. These results may demonstrate that the annealing process introduced the microphase separation. MWCNTs, dispersed in a soft segment of PU-Ether, were oriented and consequently obstructed the mobility of flexible chains.

### 3.3. Morphology

**Figure 5:**
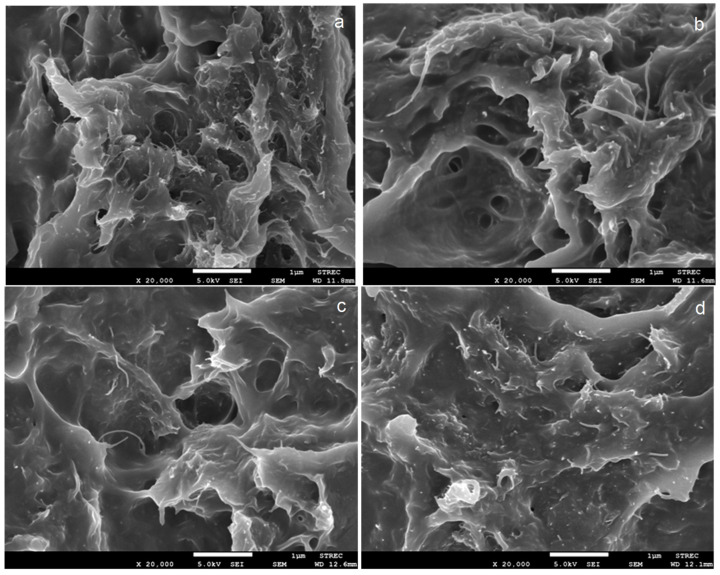
FESEM images of PU-Ester/MWCNT nanocomposites unannealed (**a**) and annealed at 80 °C (**b**), 100 °C (**c**) and 120 °C (**d**) for 6 h.

**Figure 6:**
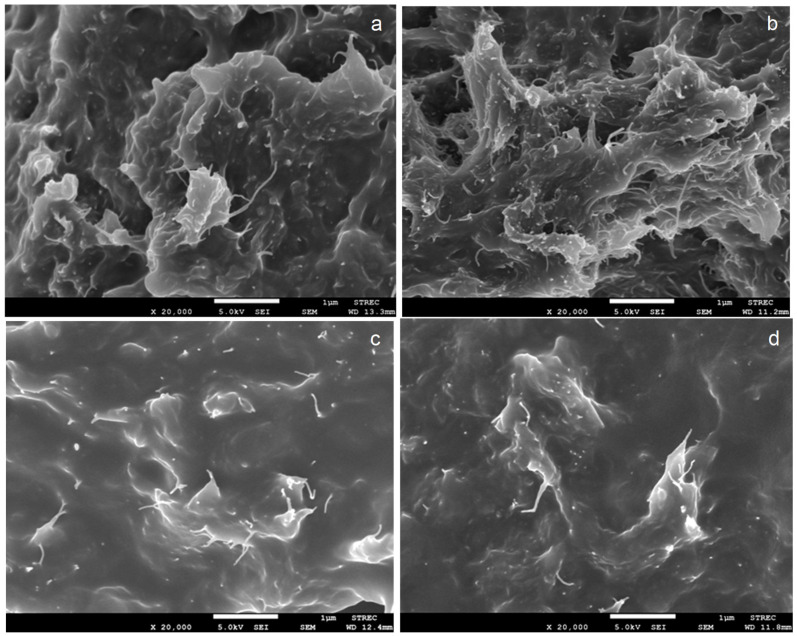
FESEM images of PU-Ether/MWCNT nanocomposites unannealed (**a**) and annealed at 80 °C (**b**), 100 °C (**c**) and 120 °C (**d**) for 6 h.

The fracture surfaces of the tensile-testing specimens were characterized by FESEM to investigate MWCNT dispersion after annealing. FESEM images of PU nanocomposites, unannealed and annealed at 80 to 120 °C for six hours, are shown in Figure 5 and Figure 6. The fracture surfaces of all nanocomposites were rough. This implies that the annealing process did not affect the toughness of PU nanocomposites based on different soft segments. MWCNTs were able to disperse in PU matrices with good distribution and dispersion. It was found that the annealing temperature and time did not affect the agglomeration of MWCNTs in either PU-Ester (Figure 5) or PU-Ether matrices (Figure 6). Nanocomposites of PU, based on different soft-segment structures, showed similar behaviors. Moreover, no significant difference in MWCNT dispersion was observed between unannealed and annealed nanocomposites. Thermal treatment could affect the movement of polymer chains, resulting in the development of amorphous and crystalline parts. However, MWCNTs were still well dispersed in segmented PU with increasing annealing temperatures.

### 3.4. Conductivity

**Figure 7:**
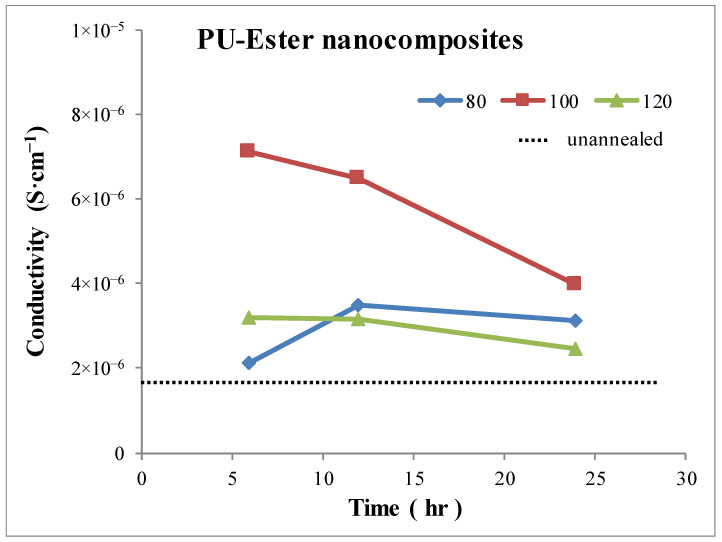
Conductivities of PU-Ester /MWCNT nanocomposites with various annealing conditions.

**Figure 8:**
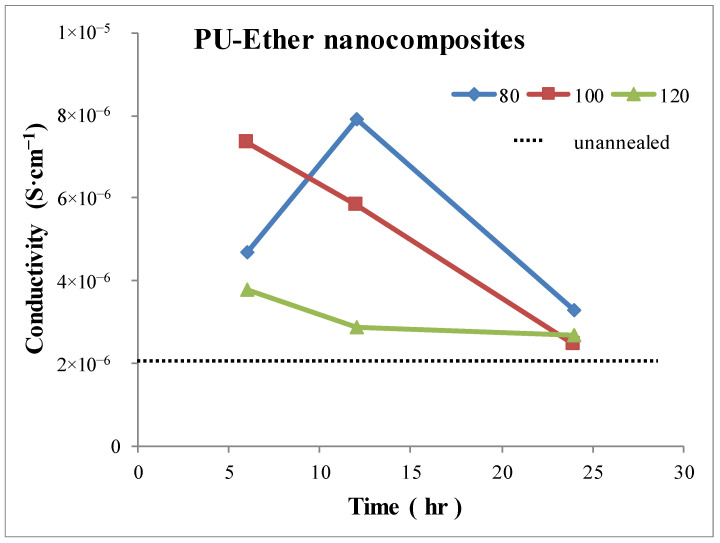
Conductivities of PU-Ether /MWCNT nanocomposites under various annealing conditions.

The volume conductivities of nanocomposites were determined. Measurements were performed in the frequency range of 20–1 × 10^6^ Hz in order to detect the frequency range in which the conductivities of all the MWCNT nanocomposites remained constant. These persistent conductivities at the lowest frequency were collected and subsequently analyzed so as to gain an understanding of the effect of the annealing process. After determining the conductivity values using a variety of frequencies, conductivities of 200 Hz were used for all the nanocomposites. The conductivities of PU-Ester and PU-Ether nanocomposites are shown in Figure 7 and Figure 8, respectively. The conductivity of unannealed PU-Ester nanocomposites was found to be 1.76 × 10^−6^ S·cm^−1^ and that of unannealed PU-Ether nanocomposites was 2.14 × 10^−6^ S·cm^−1^. After heat treatment under different conditions, all the annealed nanocomposites demonstrated an improvement in conductivity. Conductivity decreased with longer annealing time, excluding at the annealing temperature of 80 °C. Similar results were found for nanocomposites of PU based on different soft-segment structures.

Considering the PU-Ester/MWCNT nanocomposites in Figure 7, the annealing temperature of 100 °C had higher conductivity. According to the DSC results, the segregated hard-segment phase tended to crystallize at an annealing temperature over 100 °C. This implies that the crystallization accommodated the movement of MWCNTs to form the conductive network. However, a more ordered structure of the hard domain may have disrupted the formation of the MWCNT network, resulting in the decrease in conductivities of the PU-Ester nanocomposites annealed at 120 °C. Therefore, the highest conductivity of 7.12 × 10^−6^ S·cm^−1^ was observed at an annealing temperature of 100 °C for 6 h.

On the other hand, the microphase separation of the hard segment was found at 80–100 °C and the ordered structure of the segregated hard phase was introduced at an annealing of 120 °C, as demonstrated by the thermal characterization results for the PU-Ether nanocomposites. The effect of the annealing condition was, therefore, different from that of PU-Ester nanocomposites. The improvement in the conductivity of PU-Ether nanocomposites annealed at 80 and 100 °C seemed better than those subjected to an annealing temperature of 120 °C. This implies that the crystalline structure of the segregated hard domain in PU-Ether could limit the soft-segment mobility and MWCNT network arrangement. PU-Ether/MWCNT nanocomposites, annealed at 80 °C for 12 h, were found to have the highest conductivity, at 7.90 × 10^−6^ S·cm^−1^.

## 4. Conclusions

The effect of annealing conditions on the selective dispersion of MWCNTs was studied. Annealed PU/MWCNT nanocomposites, based on two different soft segments (i.e., polyester and polyether), varied by temperature and time, between 80 and 120 °C, and 6 and 24 h. Microphase separation was found to increase and the crystallinity of the hard segment after heat treatment was observed in both thermoplastic PU matrices. Glass transition temperatures were determined for hard segments and the multiple melting endotherms. These results were the evidence of the appearances of the microphase separation and the crystallinity of hard segment. The effects of annealing process were investigated; firstly, accommodated the segregated hard segment domain to align amorphous form and subsequently, crystallized with the annealing temperature increase. As the result of selective dispersion of MWCNT in different segregated domains, the effects of annealing conditions on mechanical property were diversified. MWCNTs dispersed in PU-Ether soft segments, resulting in the significant decrease in elongation. However, there was no noticeable decline in the elasticity of PU-Ester nanocomposites caused by MWCNTs dispersing in the hard domain. The effect of annealing temperature on microphase separation morphology was investigated. PU-Ester nanocomposites annealed at 100 and 120 °C showed crystallinity in the hard-segment domain. On the other hand, PU-Ether nanocomposites annealed at 80 and 100 °C introduced microphase separation, and the segregated hard domain aligned in amorphous form. Conductivity was also affected by the annealing process. Annealed nanocomposites of PU-Ester with a hard-segment crystalline structure accommodated the improvement in conductivity, although the increase in crystallinity could have caused the disruption of the MWCNT network formation. Therefore, the highest conductivity value (7.12 × 10^−6^ S·cm^−1^) was annealed for PU-Ester nanocomposites at 100 °C for 6 h. In contrast, PU-Ether/MWCNT nanocomposites were found to have better conductivity under the annealing conditions in which hard segments formed an amorphous morphology. Consequently, PU-Ether nanocomposites annealed at 80 °C for 12 h had the highest conductivity, at 7.90 × 10^−6^ S·cm^−1^.

## Figures and Tables

**Table 1:** DSC analysis of PU-Ester/MWCNT nanocomposites and PU-Ether/MWCNT nanocomposites.

Sample	T_g(SP)_	T_g(MP)_	T_g(HP)_	T_MM(L)_ (°C)			T_MM(H)_	∆H_MM_
	(°C)	(°C)	(°C)	A	B	C	(°C)	(J·g^−1^)
Es-unannealed	−30	69	-	-	165	-	191	14.9
Es-80-6	−28	62	99	-	166	-	191	10.7
Es-80-12	−30	60	102	-	164	-	190	10.3
Es-80-24	−28	61	102	-	167	-	192	9.9
Es-100-6	−31	63	-	125	161	-	192	19.6
Es-100-12	−31	62	-	127	165	-	189	20.3
Es-100-24	−30	62	-	128	166	-	190	21.5
Es-120-6	−33	65	-	145	166	-	189	17.4
Es-120-12	−33	63	-	145	167	-	191	16.7
Es-120-24	−32	64	-	149	167	-	192	17.1
Et-unannealed	-	74	-	-	-	-	196	14.9
Et-80-6	-	67	100	-	167	177	199	15.3
Et-80-12	-	65	98	-	161	175	196	15.9
Et-80-24	-	65	100	-	166	178	199	11.8
Et-100-6	-	68	119	-	166	177	198	12.0
Et-100-12	-	68	119	-	163	-	197	11.1
Et-100-24	-	66	122	-	165	177	198	10.9
Et-120-6	-	68	-	145	161	-	196	19.9
Et-120-12	-	68	-	148	168	-	199	18.8
Et-120-24	-	62	-	152	169	180	201	19.3

## Data Availability

The data presented in this study are available on request from the corresponding author.
